# B7-H4 enhances the differentiation of murine leukemia-initiating cells via the PTEN/AKT/RCOR2/RUNX1 pathways

**DOI:** 10.1038/leu.2017.232

**Published:** 2017-08-15

**Authors:** F Xia, Y Zhang, L Xie, H Jiang, H Zeng, C Chen, L Liu, X He, X Hao, X Fang, X Liu, F Zhang, H Gu, J Wan, Y Cheng, C C Zhang, G-Q Chen, Y Lu, Z Yu, J Zheng

**Affiliations:** 1 Department of Pathophysiology, Hongqiao International Institute of Medicine, Shanghai Tongren Hospital/Faculty of Basic Medicine, Key Laboratory of Cell Differentiation and Apoptosis of Chinese Ministry of Education, Shanghai Jiao Tong University School of Medicine, Shanghai, China; 2 Institute and Department of Endocrinology and Metabolism, Shanghai Ninth People’s Hospital, Shanghai Jiao Tong University School of Medicine, Shanghai, China; 3 Department of Hematology, Xinhua Hospital, Shanghai Jiao Tong University School of Medicine, Shanghai, China; 4 Department of Hematology, Shanghai Ninth People’s Hospital, Shanghai Jiao Tong University School of Medicine, Shanghai, China; 5 Departments of Physiology and Developmental Biology, UT Southwestern Medical Center, Dallas, TX, USA

Leukemia-initiating cells (LICs) are believed to be responsible for the initiation, development and relapse of leukemia.^1^ To effectively eliminate LICs, new molecular targets and therapeutic strategies for targeting either extracellular or intracellular signaling are needed. Studies from our and other groups have suggested that targeting specific surface immune molecules of LICs, including LILRB2,^2^ CD123,^3^ CD47^4^ and CD93,^5^ may be a promising strategy to block leukemogenesis. To identify more such surface immune molecules, we screened approximately 30 cell surface proteins expressed in immune systems, and found that several immune molecules, including IREM-1, BTLA, CD244, JAM3, B7-H1 and B7-H4, were highly expressed on MLL-AF9-induced human acute myeloid leukemia (AML) cells.^6^ Interestingly, one of the identified candidates B7-H4 was also expressed on one fraction of human LIC-enriched CD34^+^ AML cells as determined by flow cytometric analysis (Supplementary Figure 1a). More importantly, *in silico* analysis using data extracted from the curated database Leukemia Gene Atlas (http://www.leukemia-gene-atlas.org) also showed that B7-H4 expression level was positively correlated with the overall survival of AML patients (Supplementary Figure 1b), indicating that B7-H4 may serve as a tumor suppressor during leukemia development.

B7-H4 is initially found to be expressed on the surfaces of antigen-presenting cells and negatively regulates the activities of T cells or neutrophils by binding to an unknown receptor.^7^ However, the role of B7-H4 in cancer development remains controversial since B7-H4 has been shown to either enhance^8^ or inhibit tumorigenesis^9^ through the evasion of immune surveillance or other unknown mechanisms. To determine the function of B7-H4 in LICs, we used B7-H4 knockout (KO) mice to establish an MLL-AF9-induced AML model and evaluated its role in the development of AML.

As expected, the B7-H4 expression level was only detected on the wild-type (WT), but not B7-H4-null leukemia cells (Supplementary Figure 1c). Interestingly, the frequencies of B7-H4-null YFP^+^ leukemia cells in the peripheral blood are slightly higher than those in WT controls upon primary transplantation although there was no significant difference (Supplementary Figures 1d and e). We also observed a minor reduction in the survival of the primary B7-H4-null recipients (57.5 vs 69 days, Figure 1a, first panel), indicating that B7-H4 may suppress leukemogenesis. Strikingly, there were much higher percentages of B7-H4-null YFP^+^ donor cells in the peripheral blood in the second and third transplantation (Supplementary Figures 1d and e), and survival was also markedly decreased in recipients receiving B7-H4-null donor cells upon the subsequent second and third transplantation compared to those of WT controls (20.5 vs 33, 19.5 vs 28 days, respectively, Figure 1a, second to third panel). More importantly, limiting dilution assays with YFP^+^ leukemia cells isolated from the second transplant revealed that the LIC frequency was 1 in 20 of B7-H4-null leukemia cells, which was ~10-fold higher than that in WT controls (1 in 198) (Figure 1a, fourth panel). However, B7-H4 deletion did not affect the development of T-cell acute lymphoblastic leukemia induced by activated Notch1 during the serial transplantation (Supplementary Figures 2a–g). These results strongly indicate that B7-H4 may act as a tumor suppressor specifically for AML and does not affect functions of hematopoietic stem cells as we previously described,^10^ which suggests that B7-H4 may be an ideal target for the elimination of LICs.

To further elucidate the potential mechanisms leading to the enhanced proliferation of LICs, Wright–Giemsa staining was conducted and revealed that there was a much higher number of immature B7-H4-null blast cells in the bone marrow (BM) compared to that of the WT counterparts (36.7±3.4 vs 18.5±1.6%, Supplementary Figure 3a), consistent with the increased infiltration of B7-H4-null leukemia cells in the spleen and liver as indicated by the changes of relative weights and hematoxylin/eosin staining (Supplementary Figures 3b and c). Consistently, the frequency of B7-H4-null Mac-1^+^Gr-1^−^ leukemia cells (Gr-1 expression level indicates the extent of myeloid cell differentiation) in the peripheral blood increased markedly to twofold greater than that in WT controls upon primary transplantation (38.7±1.2% vs 17.2±3.4%, Figure 1b). Consistent with *in vivo* data, B7-H4-null LICs had marked decreased ability to differentiate to Mac-1^+^Gr-1^+^ mature leukemia cells *in vitro* upon stimulation by phorbol 12-myristate 13-acetate than WT counterparts (Supplementary Figure 3d). Moreover, the subsequent secondary and tertiary transplantations revealed similar changes in the differentiation status of B7-H4-null leukemia cells and displayed as a marked elevation in the percentage of the Mac-1^+^Gr-1^−^ cell population (Figure 1b).

To ask how B7-H4 influences the differentiation of LICs, we performed messenger RNA-sequencing analysis using WT and B7-H4-null Mac-1^+^c-Kit^+^ LICs. Interestingly, the mRNA-sequencing data revealed that several pathways other than immune regulation, including transcription (most markedly changed) and chromosome and chromatin organization, were also markedly altered (Supplementary Figure 4a). Several candidate genes related to transcription factors that may be important for the stemness of cancer stem cells (Rcor2, Klf2, Klf4, Klf6 and Hif-1α) and LIC differentiation (Cebpa, Gata2 and Pu.1), but not for self-renewal, were notably changed in B7-H4-null LICs (Supplementary Figures 4a and b). The expression levels of some potential targets (Rcor2, Klf4, Cebpa, Gata2, Runx1 and Hif-1α) were further determined using quantitative reverse transcription-polymerase chain reaction (Figure 1c, first panel and Supplementary Table 1), which showed a significant increase in B7-H4-null LICs than that in WT counterparts.

Rcor2 has been involved in multiple functions, including cell differentiation induction, self-renewal maintenance and epigenetic modifications in different cell types,^11, 12, 13^ which promotes us to test its potential connections with the phenotypes in B7-H4-null leukemic mice. Intriguingly, we revealed that the RCOR2 protein level was substantially elevated in B7-H4-null AML cells (Figure 1c, second panel). B7-H4-null AML cells were then treated with Rcor2-targeting short hairpin RNA (shRcor#2, Supplementary Table 1) followed by the assessment of the *in vivo* engraftment. The mice transplanted with the Rcor2-knockdown B7-H4-null AML cells developed leukemia much more slowly than that of mice transplanted with B7-H4-null leukemia cells, which was comparable to their WT counterparts (Figure 1c, third panel and Supplementary Figure 4d). These results demonstrate that B7-H4 inhibits leukemogenesis via downregulation of RCOR2.

Because we also observed that RUNX1 was highly upregulated in B7-H4-null LICs (Figure 1c), which has been reported to maintain the stemness of both hematopoietic stem cells and LICs in a dose-dependent manner,^14, 15^ this promoted us to examine whether RCOR2 directly controls the expression of RUNX1 to inhibit the differentiation of B7-H4-null LICs. We thus ectopically expressed or knocked down Rcor2 in a mouse AML cell line (C1498) and observed that RUNX1 level was notably upregulated or reduced (Figure 2a, first to second panel). In addition, we further demonstrated that Rcor2 could directly transactivate Runx1 as evaluated by using a luciferase reporter (pGL3-Runx1, Supplementary Figure 5a) or a chromatin immunoprecipitation analysis (Supplementary Figure 5b and Supplementary Table 1). Consistently, RUNX1 and RCOR2 protein levels were markedly upregulated in B7-H4-null AML cells than those of WT controls (Figure 2a, third panel). To rule out the connections between B7-H4 and RCOR2, we examined several signaling pathways that might be regulated through B7-H4 and eventually observed that serine/threonine kinase 1 (AKT) signaling was notably strengthened in B7-H4-null AML cells (Figure 2b, first panel). Interestingly, RCOR2 level was indeed significantly reduced after treatment with an AKT-specific inhibitor (MK2206) for 72 h (Figure 2b, second panel). Furthermore, we revealed that HIF-1α could directly bind to the promoter of Rcor2 as determined by a luciferase reporter (Figure 2b, third panel). Consistently, both messenger RNA and protein levels of HIF-1α were remarkably upregulated in B7-H4-null AML cells (Figure 1c and Figure 2b, first panel). Inhibition of AKT signaling by MK2206 also reduced the HIF-1α and ROCR2 levels, indicating HIF-1α is one of the transcription factors involved in the AKT-mediated ROCR2 activation (Figure 2b, second panel).

Because phosphatase and tensin homolog deleted on chromosome 10 (PTEN) is the major negative regulator of AKT phosphorylation, we also examined its levels in WT and B7-H4-null AML cells, and showed that PTEN was markedly downregulated in B7-H4-null leukemia cells (Figure 5c, first panel). Considering that B7-H4 contains only two amino acids in the intracellular domain,^7^ we hypothesized that B7-H4 may be present in the cytoplasm or nucleus to regulate PTEN level. Indeed, B7-H4 existed in both the cytoplasm and nucleus as determined by immunoblotting (Supplementary Figure 5c). Immunofluorescence staining with either B7-H4-over-expressing C1498 cells or mouse AML cells also showed that B7-H4 could be detected on the cell membrane, in the cytoplasm or in the nucleus (Supplementary Figures 5d and e). Interestingly, when B7-H4 was pulled down in PTEN-/B7-H4-over-expression 293T cells, both ectopically expressed PTEN and endogenous AKT could be easily detected in the immunoprecipitated cytoplasmic or nuclear samples, especially in cytoplasm (Figure 2c, second panel and Supplementary Figure 5f). Meanwhile, both ectopically expressed AKT and endogenous PTEN were clearly observed when B7-H4 was pulled down from AKT-/B7-H4-over-expressing 293T cells (Figure 2c, third panel). Furthermore, both overall level of ubiquitination and endogenous PTEN ubiquitination was markedly decreased following the overexpression of B7-H4 (Figure 2c, fourth panel), indicating that it has additional functions in the regulation of ubiquitination, although B7-H4 did not affect the transactivation of PTEN (Supplementary Figure 5g).

RCOR2 is usually considered a transcriptional repressor for the maintenance of the self-renewal of embryonic stem cells, neural stem cells.^12^ Surprisingly, our study showed that RCOR2 enhances the expression of RUNX1, indicating that this protein serves as a co-activator in LICs. Further studies are required to delineate how RCOR2 shifts between a transcriptional repressor and a coactivator and to determine how this protein controls the expression levels of RUNX1. Herein, we present the first evidence that B7-H4 acts as a tumor suppressor to through PTEN/AKT/HIF-1α/RCOR2/RUNX1 pathways to promote the differentiation of LICs, but does not impair the normal functions of hematopoietic stem cells (Supplementary Figure 5h). B7-H4 expression level is positively correlated with the overall survival of AML patients. Activation of B7-H4 by functional antibodies or other small molecule chemicals may be an efficient way to eliminate LICs. These results also indicate targeting LICs through its downstream molecules, such as RCOR2, may be other valuable strategies for the treatment in leukemia.

## Figures and Tables

**Figure 1:**
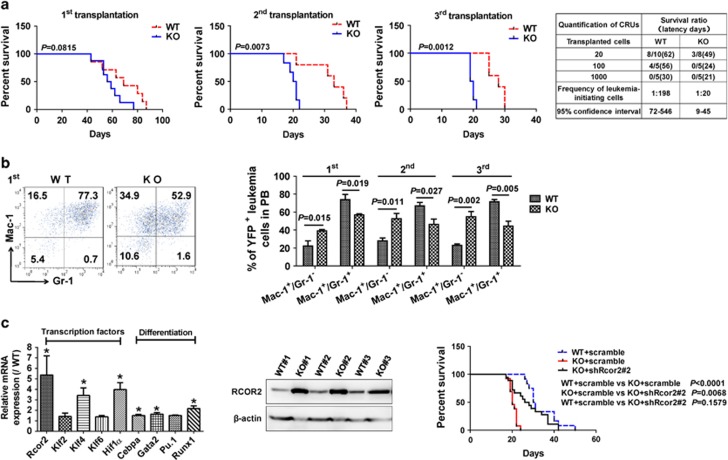
B7-H4 promotes the differentiation of LICs to inhibit leukemogenesis by downregulating the RCOR2 level. (**a**) Survival was compared between the recipients transplanted with MLL-AF9-infected WT and B7-H4-null (KO) hematopoietic stem/progenitors upon primary transplantation (first, *n*=7–8, log-rank test, first panel), and mice receiving 10 000 WT or B7-H4-null YFP^+^ door leukemia cells from the primary (second, second panel) and secondary (third, third panel) recipients (*n*=5–6, log-rank test). Limiting dilution assays comparing the frequencies of LICs in WT and B7-H4-null BM cells. The indicated numbers of YFP^+^ leukemia cells isolated from secondary recipients were co-injected with 2 × 10^5^ competitor cells into lethally irradiated recipients (fourth panel). The competitive repopulating units (CRUs) were evaluated using L-Calc software and indicated. (**b**) Representative flow cytometric analysis of the YFP^+^Mac-1^+^Gr-1^+^ and YFP^+^Mac-1^+^Gr-1^-^ leukemia cells in the peripheral blood of primary recipients receiving transplants of MLL-AF9-transduced WT or B7-H4-null hematopoietic stem/progenitors (first panel), and qualification of frequencies of YFP^+^Mac-1^+^Gr-1^+^ and YFP^+^Mac-1^+^Gr-1^−^ leukemia cells in the peripheral blood (PB) of primary, secondary and tertiary recipients are shown (*n*=5, second panel). (**c**) Potential candidate genes related to transcription factors (important for stemness) and myeloid differentiation were validated in fluorescence activated cell sorting-purified WT or B7-H4-null YFP^+^Mac-1^+^c-Kit^+^ LICs using quantitative Reverse transcription-polymerase chain reaction analysis (*n*=3, first panel), and protein levels of RCOR2 in WT and B7-H4-null BM AML cells were measured using immunoblotting (*n*=3, second panel). Rcor2 was silenced in B7-H4-null AML cells using shRcor2#2, which were transplanted into the recipient mice. The survival was analyzed among the mice receiving WT cells, B7-H4-null cells and Rcor2-knockdown B7-H4-null cells (*n*=12–18, log-rank test, third panel). mRNA, messenger RNA.

**Figure 2:**
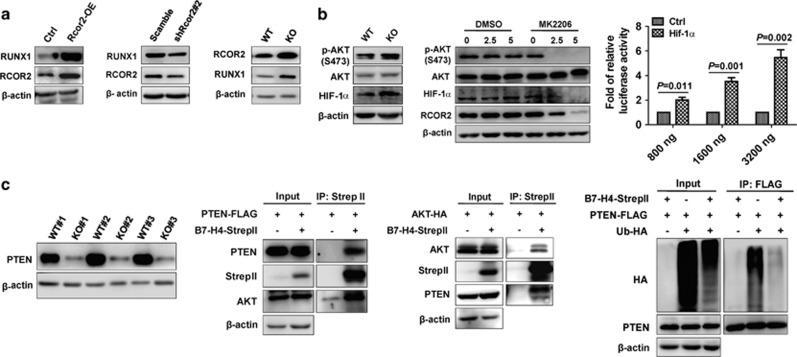
B7-H4 suppresses RCOR2 to reduce RUNX1 expression through PTEN/AKT signaling. (**a**) Protein levels of RUNX1 and RCOR2 were evaluated in Rcor2-over-expressing C1498 cells (first panel), Rcor2-knockdown C1498 cells (second panel) and WT and B7-H4-null BM leukemia cells (third panel) using immunoblotting. (**b**) Expression levels of p-AKT, AKT and HIF-1α were measured in the WT and B7-H4-null BM AML cells (first panel). BM AML cells were treated with the AKT inhibitor MK2206 at the indicated doses (0, 2.5 and 5 μM) for 72 h followed by the evaluation of the protein levels of p-AKT, AKT, HIF-1α and RCOR2 using immunoblotting (second panel). Luciferase activity was measured in 293T cells transfected with a luciferase reporter containing the Rcor2 promoter (−1770–719) and indicated doses of HIF-1α plasmid, and normalized by Renilla. The relative luciferase activity of HIF-1α were further standardized to that of control vector (*n*=6, third panel). (**c**) Protein levels of PTEN were measured in WT and B7-H4-null AML cells using immunoblotting (first panel). StrepII-tagged B7-H4 and Flag-tagged (a specific tag with eight amino acids for detection) PTEN were overexpressed in 293T cells, and their lysates were co-immunoprecipitated using strep-tactin sepharose followed by western blotting analysis for Flag (PTEN) and endogenous levels of AKT (second panel). StrepII-tagged B7-H4 and HA-tagged AKT were overexpressed in 293T cells, and their lysates were co-immunoprecipitated (IP) using strep-tactin sepharose followed by western blotting analysis for HA (AKT) and endogenous levels of PTEN (third panel). StrepII-tagged B7-H4, Flag-tagged PTEN and HA-tagged ubiquitin were overexpressed in 293T cells followed by the addition of the proteasome inhibitor MG132 in culture during the last 4 h. The lysates were immunoprecipitated using an anti-Flag M2 affinity gel followed by western blotting for HA (ubiquitin) (fourth panel).
