# Supplementary material for: B7-H4 enhances the differentiation of murine leukemia-initiating cells via the PTEN/AKT/RCOR2/RUNX1 pathways
Source: Leukemia. 2017 Aug 15;31(10):2260–4. doi: 10.1038/leu.2017.232 (PMC5629360; doi:10.1038/leu.2017.232)
Supplement: Supplementary Information [file leu2017232x1.pdf]

Materials and methods

Mice

The B7-H4 knockout mice with a C57BL/6 background were kindly provided by Dr. Lieping Chen at Yale University (Baltimore, MA, USA). The C57BL/6 mice were purchased from the Shanghai SLAC Laboratory Animal Co. Ltd. All animals were housed under specific pathogen-free conditions at a laboratory animal care-approved facility at Shanghai Jiao Tong University School of Medicine, China. All animal experimental procedures were approved based on the Guidelines for Central Animal Care and Use of the Committee of Shanghai Jiao Tong University School of Medicine.

Retroviral infection and transplantation

The MSCV-MLL-AF9-IRES-YFP (for AML model, Nature, 2006, 818-822)- or Notch1-IRES-GFP- (for T-ALL model, Science, 2004, 269–271) encoding plasmids and pCL-ECO packaging plasmid were used for the preparation of retroviruses as previously described (J clin invest., 2016, 4537-4553). Briefly, the MLL-AF9 or Notch1 and pCL-ECO (2:1 ratio) plasmids were co-transfected in 293T cells (obtained from Cell Resource Center of Shanghai Institute for Biological Science, Chinese Academy Science, Shanghai, China), and the retroviruses were harvested 48-72 h later. Lin^- fetal liver cells were purified from WT and B7-H4-null mice using AutoMACS followed by two rounds of spinoculation. A total of 2-3x10^5 infected Lin^- fetal liver cells were injected into lethally irradiated (10 Gy) C57BL/6 mice through retro-orbital injection. For secondary and tertiary transplantation, a total of 1x10^4 of YFP^+ BM leukemia cells from primary and secondary recipients were sorted using flow cytometry followed by transplantation into recipients. For the rescue experiments, the lentiviral plasmid pGIPZ-shRcor2-GFP (targeting murine Rcor2, sTable 1) and packaging plasmids pSPAX2 and pMD2G (4:3:1) were transfected in 293T cells using a calcium phosphate transfection method. The resulting lentiviral supernatant was harvested for the spin infection with B7-H4-null bone marrow (BM) leukemia cells followed by retro-orbital injection into the recipients. For each group, at least 5 recipient mice (usually 8-10-week old) were randomly and blindly divided into 2-3 groups and used

for the transplantation. All the experiments were repeated at least 3 times. In another experiment, Rcor2 was silenced in C1498 cells, an AML cell line, using Rcor2-targeting shRNA followed by the analysis of RUNX1 protein level using western blotting.

Flow cytometry

Peripheral blood or BM cells were collected through retro-orbital bleeding or isolated from the femurs and tibias of leukemic mice. The lineages and immunophenotypic $YFP^+Mac\text{-}1^+c\text{-}Kit^+$ LICs were analyzed using a combination of antibodies, including anti-mouse Mac-1-APC (Miltenyi Biotec, catalog number: 130-098-088), anti-mouse Gr-1-PE (eBioscience, catalog number:12-5931-83), anti-mouse CD3-APC (BD, catalog number: 553066), anti-mouse B220-PE (eBioscience, catalog number: 12-0452-85) and anti-mouse c-Kit-PE monoclonal antibodies (eBioscience, catalog number: 12-1171-83). For the detection of B7-H4 expression on the $CD34^+$ cells of AML patients, anti-human CD34-APC (Miltenyi Biotec, catalog number: 130-090-954 , anti-human B7-H4-biotin (eBioscience, catalog number: 13-5949-80) and streptavidin-FITC (secondary antibody) were used (eBioscience, catalog number: 11-4317-87).

Western blotting and co-immunoprecipitation

Nuclear and cytoplasmic fractions were extracted using Nuclear and Cytoplasmic Protein Extraction Kit (Beyotime Biotechnology), and total proteins were isolated by Radio Immune Precipitation Assay (RIPA) buffer (Beyotime Biotechnology) according to the manufacture's protocols. A combination of different plasmids of MSCV-HA-AKT-IRES-mCherry, pBabe-PTEN-3XFlag, pLVX-strepII-B7-H4-IRES-GFP and pLVX-strepII-IRES-GFP (empty vector) were transfected into 293T cells followed by co-immunoprecipitation (co-IP) for the analysis of their interactions. Whole cell lysates or cytoplasmic and nuclear fractions were electrophoresed on 8-10% sodium dodecyl sulfate polyacrylamide gels and transferred onto polyvinylidene difluoride membranes (Millipore). The membranes were blocked with 5% non-fat milk/TBS and

incubated with primary antibodies at 4°C overnight. The following antibodies were used for blotting: anti-AKT (Santa Cruz, catalog number: sc-8312), anti-phospho AKT (Ser473) (Cell Signaling Technology, catalog number: 4060s), anti-RCOR2 (Abcam, catalog number: Ab113826), anti-RUNX1 (Abways, catalog number: AY4014), anti-HA (Sigma-Aldrich, catalog number: H9658), anti-strepII (Genescript, catalog number: A00626), anti-PTEN (Proteintech, catalog number: 22034-I-AP), anti-HIF-1α (Proteintech , catalog number: 20960-I-AP), anti-Laminin B (Santa Cruz, catalog number: sc-6216), anti-β-tublin (Sigma-Aldrich,catalog number: T4026) and anti-β-actin (MBL, catalog number: PM053-7).

RNA sequencing

Total RNA was extracted from B7-H4 WT and B7-H4-null $YFP^+Mac\text{-}1^+c\text{-}Kit^+$ LICs for RNA-sequencing library construction using a standard TruSeq RNA sample preparation v2 protocol (Illumina). The samples were subsequently sequenced using the Illumina HiSeq2500 platform. Gene expression levels are represented as fragments per kilobase per million mapped reads (FPKM). The RNA-sequencing data were deposited in the Gene Expression Omnibus (GEO) repository under accession number GSE89161.

Immunofluorescence staining, Wright-Giemsa staining and hematoxylin and esosin staining

To determine the localization of B7-H4, immunofluorescence staining was performed in B7-H4-overexpression C1498 cells or primary mouse leukemia cells by using Anti-HA (Sigma-Aldrich, catalog number: H9658, for detecting ectopically expressed B7-H4) or anti-B7-H4 (Proteintech, catalog number: 12080-I-AP, for detecting endogenous B7-H4) antibodies, respectively. Wright-Giemsa staining was performed with BM leukemia cells of secondary recipient mice and B7-H4-overexpression JOSK-I cells, a human acute monocytic leukemia cell line (Cancer Res., 1986, 3067-3074) and the frequencies of blast cells were calculated according to their typical morphologies. Liver and spleen tissues were fixed in 4% paraformaldehyde

and embedded in paraffin. Sections were stained with hematoxylin and esosin for the analysis of the infiltration of leukemia cells.

Cell proliferation, differentiation and colony formation of JOSK-I cells

B7-H4-overexpression JOSK-I cells were counted at indicated time points for the evaluation of the change of cell growth. Meanwhile, JOSK-I cells were treated with 100 ng/ml of phorbol 12-myristate 13-acetate (PMA) for 48 h as previously described (Inflamm Res., 2007, 45-50; Cancer Res., 1986, 3067-3074). Myeloid differentiation was monitored by flow cytometric analysis with antibodies against human Mac-1-PE (eBioscience, catalog number: 12-0118-42) and CD14-PE (eBioscience, catalog number: 12-0149-42) or Wright-Giemsa staining. Colony forming assay of JOSK-I cells was performed as previously described (Oncogene, 2000, 3739-3749). Briefly, a total of 2500 B7-H4-overexpression JOSK-I cells or control cells were cultured in 1640 medium supplemented with 0.9 % of methylcellulose and 10% of FBS. Colonies were imaged and counted 7-10 days after plating.

AKT inhibitor treatment in primary leukemia cells and C1498 cells

Primary BM leukemia cells were cultured in StemSpan serum-free medium (StemCell Technologies) supplemented with 10 ng/ml mouse SCF (Peprotech), 10 ng/ml mouse IL-3 (Peprotech) and 10 ng/ml mouse IL-6 (Peprotech). An acute myeloid leukemia cell line, C1498 (ATCC), was cultured in RPMI 1640 medium (Gibco) supplemented with 10% FBS (Sigma-Adrich). Leukemia cells and C1498 cells were treated with an inhibitor specific for the phosphorylation of AKT (MK2206, Selleck) for 72 h. Different doses of MK2206 (0, 2.5, and 5.0 μM) were applied to suppress AKT activities, and protein levels of phosphorylated AKT, AKT, HIF-1α and RCOR2 were determined using western blotting.

Analysis for luciferase activities

To evaluate the transcriptional regulation of RUNX1 by B7-H4 or RCOR2, and PTEN by B7-H4, the promoter regions of Runx1 and Pten were incorporated into the pGL3 vector (Promega, kindly provided by Guoqiang Chen at Shanghai Jiao Tong

University School of Medicine, EMBO Rep., 2015, 1563-1580; Carcinogenesis, 2010, 1367–1375). To evaluate the transcriptional regulation of RCOR2 by HIF-1α, the promoter regions of Rcor2 were incorporated into the pGL4 vector (Promega). Cells at 50% confluence in 12-well plates were cotransfected with MSCV-HA-B7-H4-IRES-mCherry, MSCV-HA-Rcor2-IRES-mCherry, MSCV-Hif-1α-IRES-GFP, control vector, the luciferase reporter plasmids driven by promoter fragments of Runx1, Pten, Rcor2 and pRLSV40-Renilla according to different designs of experiments. Luciferases were analyzed at 24-48 h after transfection using a luciferase reporter kit (GloMax®Multi Instrument, Promega). All experiments were performed in sixtuple or triplicate. Luciferase activities were calculate by normalizing Firefly to Renilla activity (for Runx1 and Pten promoter), and compared to control vector if necessary (for Rcor2 promoter).

Chromatin immunoprecipitation assays

Chromatin immunoprecipitation (ChIP) assays were performed as previously described (Cell Stem Cell, 2010, 380-390). The 293T cells were transfected with the pLX304-Rcor2-V5 plasmids and collected for 24 h later for the cross-linking with formaldehyde at a final concentration of 1%. The samples were sonicated with a Q700 sonicator six times (5 sec on and 10 sec off for each round of sonication). RCOR2 was immunoprecipitated with anti-V5 antibodies (Biodragon Immunotechnologies Co., Ltd , B1005) and protein A/G beads (Beyotime). DNA fragments were purified using a Qiagen PCR purification kit and quantified by semi-quantitative PCR with primers for the Runx1 promoter as listed in sTable 1.

Ubiquitination assay

The 293T cells were transfected with plasmids expressing pLVX-strepII-B7-H4-IRES-GFP, pBabe-PTEN-3XFlag and CMV-ubiquitin-HA or empty vector followed by the addition of 20 μM MG132 at 4 h prior to collection. The cells were washed twice with cold PBS and subsequently lysed in 500 μL of Radio Immunoprecipitation Assay lysis buffer (RIPA, Beyotime) containing a protease

inhibitor cocktail. The lysates were immunoprecipitated with anti-Flag M2 beads (Sigma) overnight at 4 °C. PTEN ubiquitination levels were analyzed by immunoblotting with an anti-HA antibody.

Quantitative RT-PCR

Total normal BM cells, HSCs, YFP^+ leukemia cells or $YFP^+Mac\text{-}1^+c\text{-}Kit^+$ LICs were sorted using flow cytometry for the isolation of total RNA. First-strand cDNA was reverse transcribed using M-MLV reverse transcriptase (Promega, Inc.). The PCR reactions were performed according to the manufacturer's instructions. The mRNA level was normalized to the level of β-actin RNA transcripts. The primer sequences are shown in sTable 1.

Study approval

Bone marrow mononuclear cells of AML samples from the patients following diagnostic work were kindly provided by the Department of Hematology at Xinhua Hospital, the 9th People's Hospital or Tongren Hospital, Shanghai Jiao Tong University School of Medicine. Written informed consent was obtained from all of the patients and all the procedures were approved by the Ethics Committee for Medical Research (IRB) at Shanghai Jiao Tong University School of Medicine.

Statistical analysis

The data are expressed as the means± SEM. The data were analyzed using Student's t test (two-sided), and statistical significance was set at $p<0.05$. The survival rates of the two groups were analyzed using the Log-rank Test. If the mice are dead due to other unexpected reasons, but not because of the leukemia development, they will not be included for the calculation of the survival ratio. All the experiment shown was replicated 3-5 times or more.

sTable 1. Primer sequences

Gene	Sequence (5'-3')
Human B7-H4-F	GGCAGATCCTCTTCTGGAGC
Human B7-H4-R	GCATCAGGTAAGGGCTGAGA
Mouse B7-H4-F	TCAGGCAAGCACTTCATCAC
Mouse B7-H4-R	GGTCGTCTTTGCCTTCTTTG
Mouse Cebpa-F	CAAGAACAGCAACGAGTACCG
Mouse Cebpa-R	GTCACTGGTCAACTCCAGCAC
Mouse Gata2-F	CGACGAGGTGGATGTCTTCT
Mouse Gata2-R	GCTGTGCAACAAGTGTGGTC
Mouse Pu.1-F	ATGTTACAGGCGTGCAAAATGG
Mouse Pu.1-R	TGATCGCTATGGCTTTCTCCA
Mouse Runx1-F	GATGGCACTCTGGTCACCG
Mouse Runx1-R	GCCGCTCGGAAAAGGACAA
Mouse Ccnd1 -F	GCGTACCCTGACACCAATCTC
Mouse Ccnd1-R	CTCCTCTTCGCACTTCTGCTC
Mouse Rasa4-F	CGCTGTCCATCCGAATCGTAG
Mouse Rasa4-R	CTTGGTAGTCTTCACCCCAGA
Mouse Cdk5r1-F	CTGTCCCTATCCCCCAGCTAT
Mouse Cdk5r1-R	GGCAGCACCGAGATGATGG
Mouse Rcor2-F	ATCCGAGTTGGAACCAATTACC
Mouse Rcor2-F	AGTGCCTGCTCAATGTTATAGC
Mouse Klf2-F	CTCAGCGAGCCTATCTTGCC
Mouse Klf2-R	CACGTTGTTTAGGTCCTCATCC
Mouse Klf4-F	GTGCCCCGACTAACCGTTG
Mouse Klf4-R	GTCGTTGAACTCCTCGGTCT
Mouse Hif-1α F	CGGCGAGAACGAGAAGAA
Mouse Hif-1α R	AAACTTCAGACTCTTTGCTTCG
Mouse Klf6-F	GTTTCTGCTCGGACTCCTGAT
Mouse Klf6-R	TTCCTGGAAGATGCTACACATTG
Mouse β-actin-F	GGCTGTATTCCCCTCCATCG
Mouse β-actin-R	CCAGTTGGTAACAATGCCATGT
Runx1 (for ChIP)-F	GCCAGCCCGTTGTAGGGGTC
Runx1 (for ChIP)-R	ATCAAGCCGCTGCTCCGACA
Promoter-Rcor2-F	TACTCACTCGAGAAGACTCTCAGTCTTGAATC (-1770)
Promoter-Rcor2-R	TACTCAAAGCTTCAGAGGCTCCAATCCAGGAG (719)
**shRNA for Rcor2**	**Targeted sequence(5'-3')**
Mouse shRcor2#1	CAGAGCATGAAGCAGACCA
Mouse shRcor2#2	AAGAAGACAGTGATGAACT
Mouse shRcor2#3	AGCCTGGTGAAGTATTACT

sFigure 1

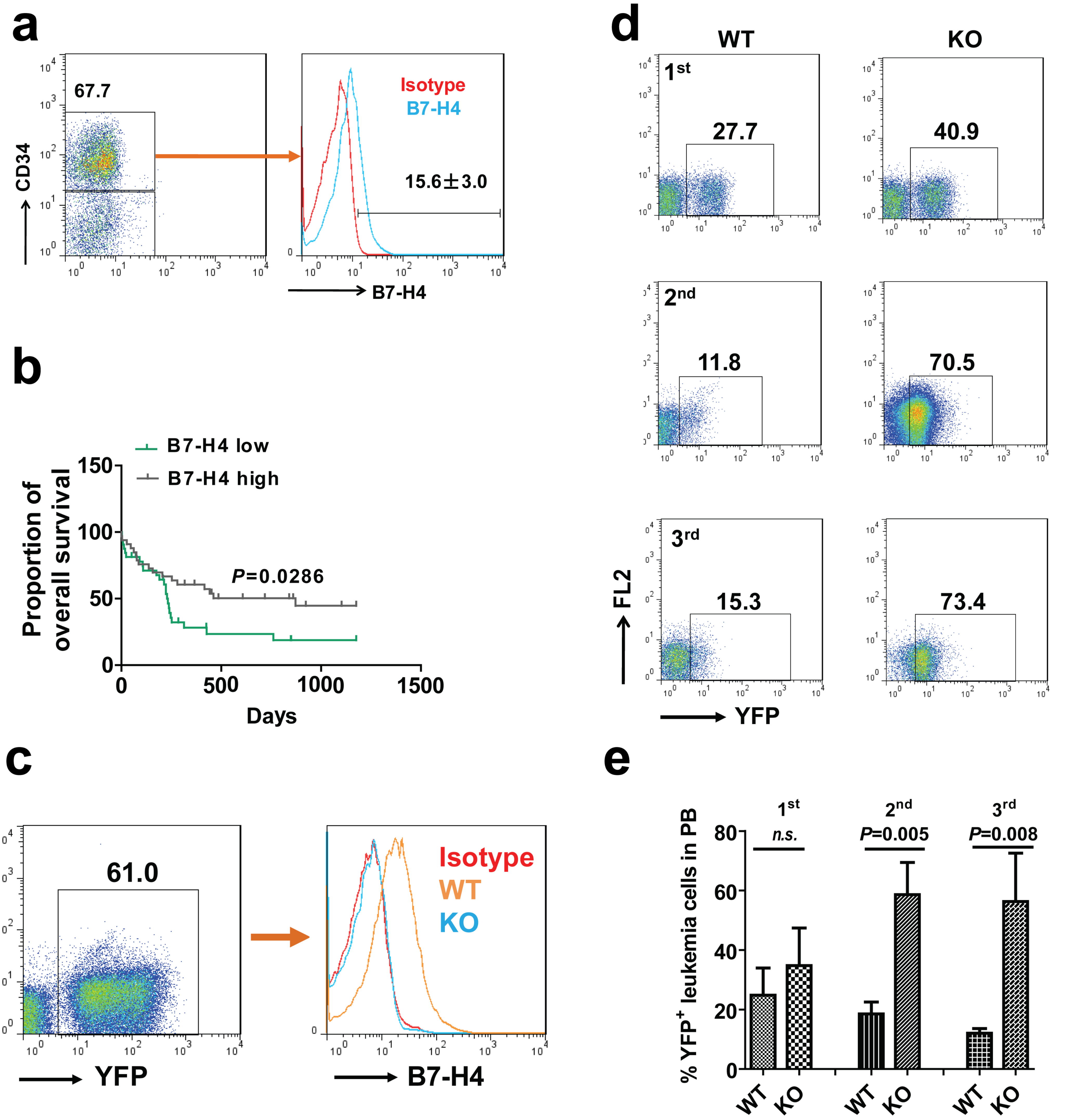
a
67.7
CD34
Isotype
B7-H4
15.6±3.0
B7-H4
b
B7-H4 low
B7-H4 high
Proportion of overall survival
P=0.0286
Days
c
61.0
YFP
Isotype
WT
KO
B7-H4
d
WT
KO
1st
27.7
40.9
2nd
11.8
70.5
3rd
15.3
73.4
FL2
YFP
e
% YFP+ leukemia cells in PB
1st
n.s.
2nd
P=0.005
3rd
P=0.008
WT
KO
WT
KO
WT
KO

sFigure 1. B7-H4 is positively correlated with the overall survival of patients and inhibits leukemogenesis in a murine AML model. a) Representative flow cytometery analysis of the expression of B7-H4 on human $CD34^+$ LIC-enriched AML cells. The frequency of $CD34^+$/$B7\text{-}H4^+$ AML cells is shown (n=7). b) In silico analysis of the relationship between the B7-H4 expression level and overall survival in AML patients (http://www.leukemia-gene-atlas.-org/LGAtlas/LGAllas.html). For each group (B7-H4 low and high), 33 patients were included for the survival analysis. c) Representative flow cytometric analysis of B7-H4 expression on WT and B7-H4-null (KO) AML cells, isotype as the negative control. d-e) Representative flow cytometric analysis for the detection of YFP^+ leukemia cells in peripheral blood of the recipients receiving transplants of MLL-AF9-transduced WT and B7-H4-null hematopoietic stem/progenitors upon primary transplantation (1st, upper panel) or $MLL\text{-}AF9^+$ WT and B7-H4-null AML cells of secondary (2nd, middle panel) and tertiary recipients (3rd, bottom panel), and quantification of the data (e, n=5-6).

sFigure 2

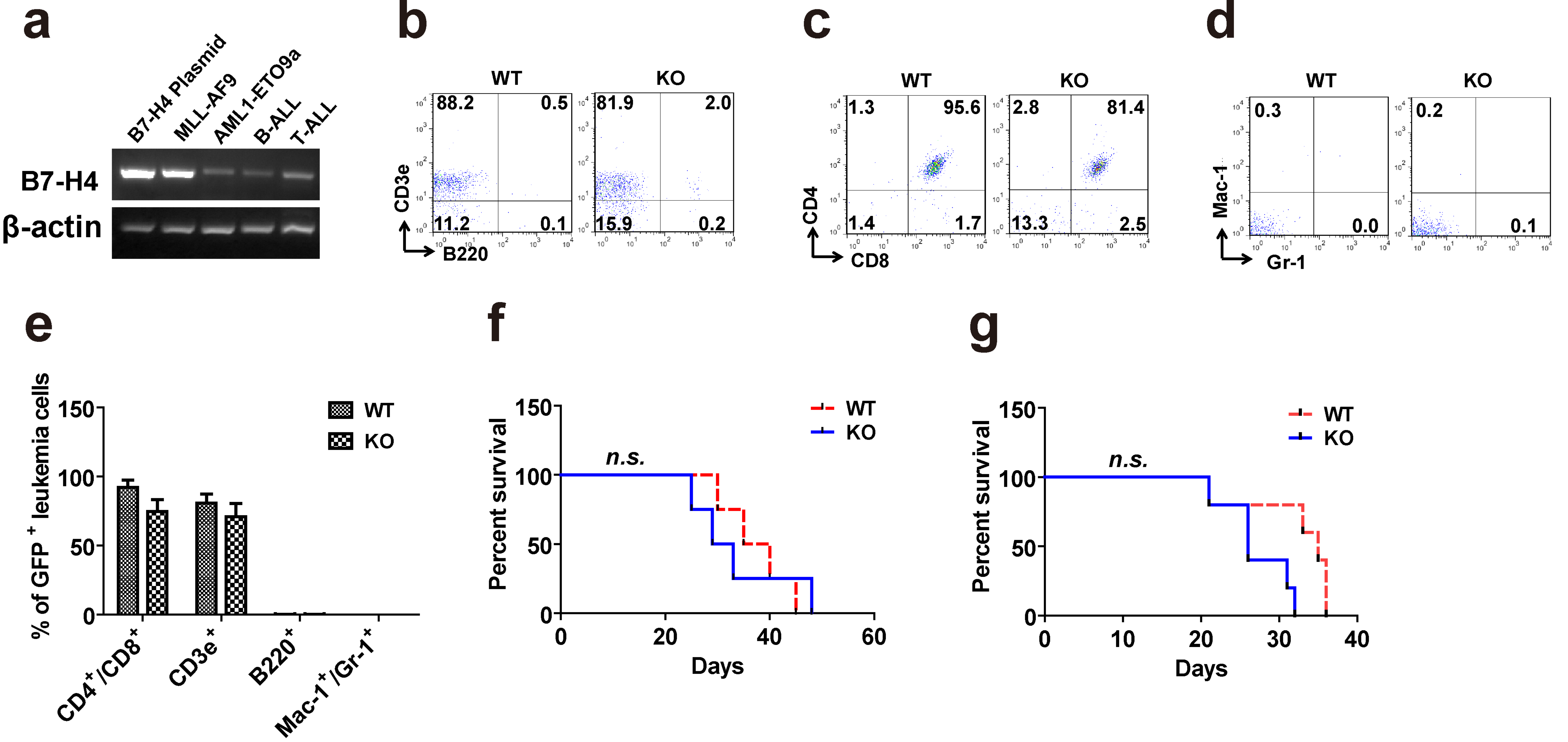
a
B7-H4 Plasmid
MLL-AF9
AML1-ETO9a
B-ALL
T-ALL
B7-H4
β-actin
b
WT
KO
88.2
0.5
11.2
0.1
81.9
2.0
15.9
0.2
CD3e
B220
c
WT
KO
1.3
95.6
1.4
1.7
2.8
81.4
13.3
2.5
CD4
CD8
d
WT
KO
0.3
0.0
0.2
0.1
Mac-1
Gr-1
e
% of GFP + leukemia cells
WT
KO
CD4+/CD8+
CD3e+
B220+
Mac-1+/Gr-1+
f
Percent survival
Days
WT
KO
n.s.
g
Percent survival
Days
WT
KO
n.s.

sFigure 2. B7-H4 has no effect in the development of T-cell acute lymphoblastic leukemia (ALL). a) Semi-quantitative RT-PCR analysis of B7-H4 levels in leukemia cells from different types of murine leukemia models, including AML1-ETO9a, N-Myc and activated Notch1 induced B-cell ALL and T-cell ALL, respectively. b-d) Representative flow cytometric plots of T-cell ALL co-stained with lymphoid cell markers (CD3/B220, b), T cell markers (CD4/CD8, c) and myeloid cell markers (Mac-1/Gr-1, d). e) Quantification results for the lineages as shown in panel b-d (n=5). f-g) Survival was compared between the recipients transplanted with activated Notch1 induced WT and B7-H4-null leukemia cells upon the primary (f) and secondary transplantation (n=4-5, log-rank test, g).

sFigure 3

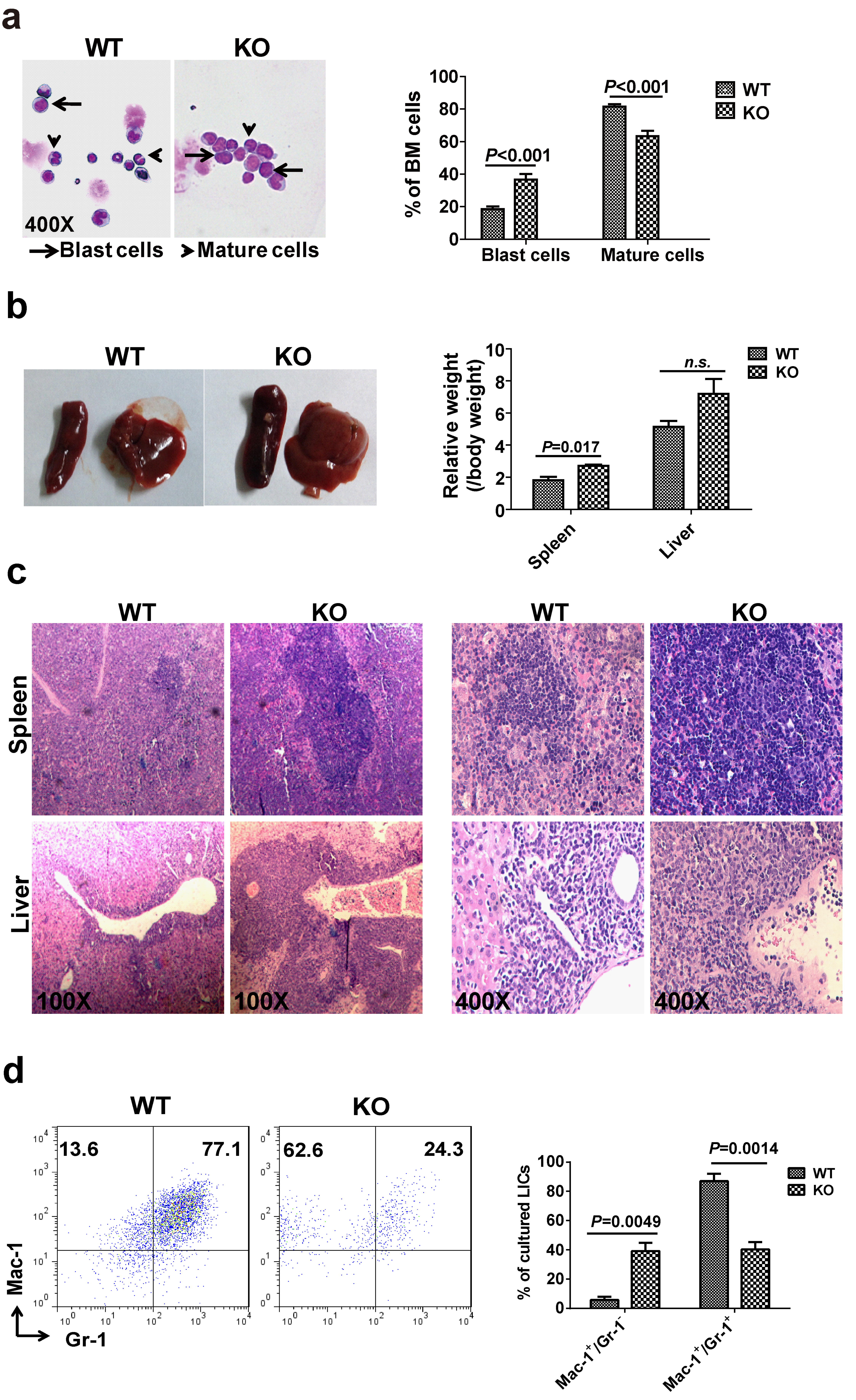
a
WT
KO
400X
Blast cells
Mature cells
100
80
60
40
20
0
% of BM cells
P<0.001
P<0.001
WT
KO
Blast cells
Mature cells
b
WT
KO
Relative weight
(/body weight)
10
8
6
4
2
0
P=0.017
n.s.
WT
KO
Spleen
Liver
c
WT
KO
WT
KO
Spleen
Liver
100X
100X
400X
400X
d
WT
KO
13.6
77.1
62.6
24.3
Mac-1
Gr-1
% of cultured LICs
100
80
60
40
20
0
P=0.0014
P=0.0049
WT
KO
Mac-1+/Gr-1-
Mac-1+/Gr-1+

sFigure 3. B7-H4 promotes the differentiation of LICs. a) Representative images of Wright-Giemsa staining with WT or B7-H4-null BM leukemia cells of primary recipients. Arrows and arrowheads indicate immature blast cells and mature leukemia cells, respectively (left panel). Quantification of the frequencies of the immature blast cells and mature leukemia cells is shown in right panel. A total of 400-500 leukemia cells from 10-15 microscope fields were counted in each sample (n=3). b) Representative sizes of spleen and liver of the primary recipients receiving WT or B7-H4-null MLL-AF9$^+$ hematopoietic stem/progenitor cells (left panel) and quantification results of normalized weights of spleens and livers of the primary recipients (right panel, n=5-7). c) Histological hematoxylin/eosin staining of AML infiltration in the spleens and livers of mice as shown in panel b. d) Representative flow cytometric analysis of the YFP$^+$Mac$^+$Gr1$^+$ and YFP$^+$Mac1$^+$Gr1$^-$ leukemia cells derived from WT or B7-H4-null YFP$^+$Mac1$^+$c-Kit$^+$ LICs upon PMA stimulation for 48 h in vitro (left panel) and quantification data are shown (right panel, n=3).

sFigure 4

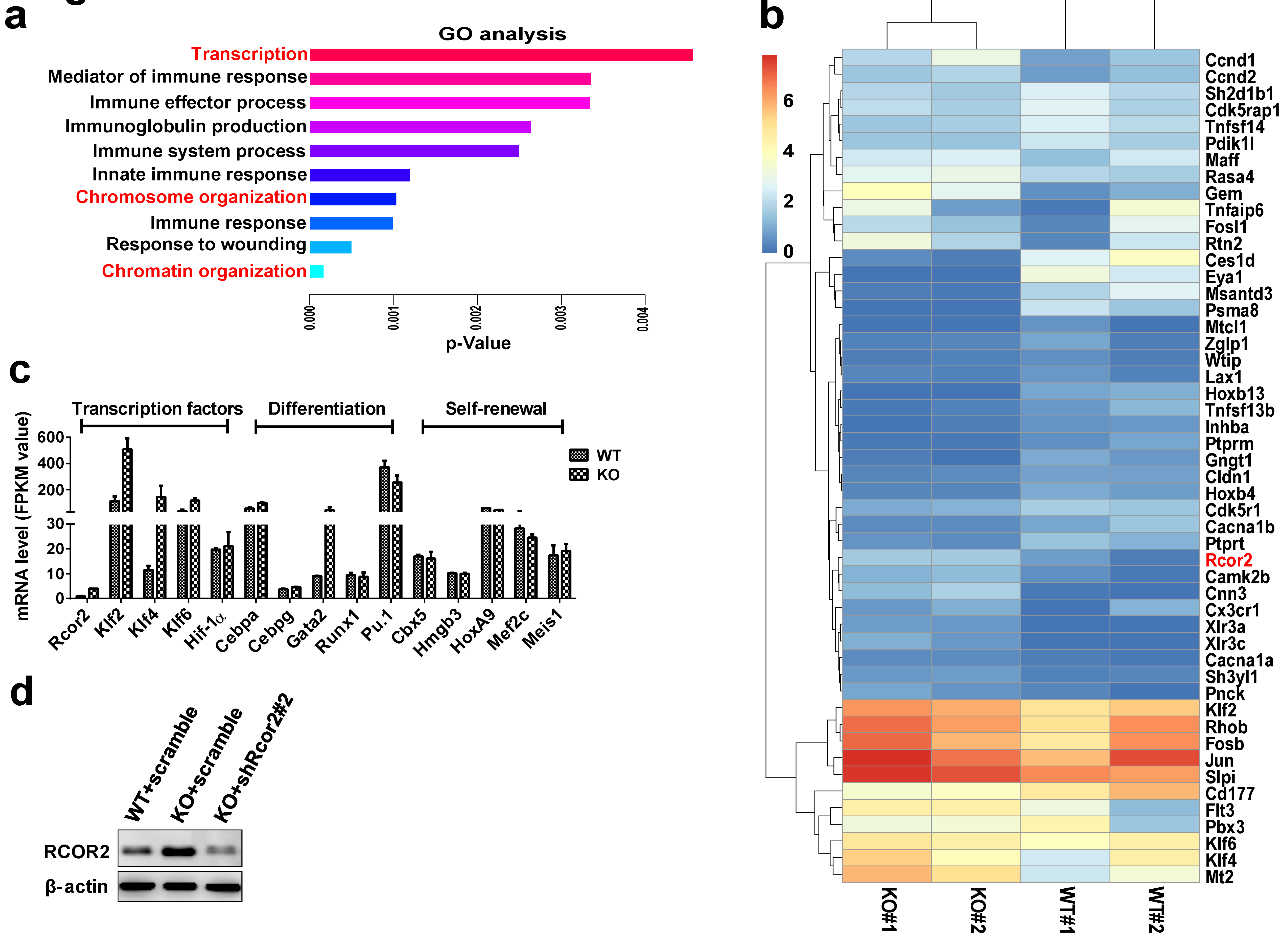
a
GO analysis
Transcription
Mediator of immune response
Immune effector process
Immunoglobulin production
Immune system process
Innate immune response
Chromosome organization
Immune response
Response to wounding
Chromatin organization
0.000
0.001
0.002
0.003
0.004
p-Value
b
6
4
2
0
Ccnd1
Ccnd2
Sh2d1b1
Cdk5rap1
Tnfsf14
Pdik1l
Maff
Rasa4
Gem
Tnfaip6
Fosl1
Rtn2
Ces1d
Eya1
Msantd3
Psma8
Mtcl1
Zglp1
Wtip
Lax1
Hoxb13
Tnfsf13b
Inhba
Ptprm
Gngt1
Cldn1
Hoxb4
Cdk5r1
Cacna1b
Ptprt
Rcor2
Camk2b
Cnn3
Cx3cr1
Xlr3a
Xlr3c
Cacna1a
Sh3yl1
Pnck
Klf2
Rhob
Fosb
Jun
Slpi
Cd177
Flt3
Pbx3
Klf6
Klf4
Mt2
KO#1
KO#2
WT#1
WT#2
c
Transcription factors
Differentiation
Self-renewal
mRNA level (FPKM value)
600
400
200
30
20
10
0
WT
KO
Rcor2
Klf2
Klf4
Klf6
Hif-1α
Cebpa
Cebpg
Gata2
Runx1
Pu.1
Cbx5
Hmgb3
HoxA9
Mef2c
Meis1
d
WT+scramble
KO+scramble
KO+shRcor2#2
RCOR2
β-actin

sFigure 4. B7-H4 suppresses leukemogenesis by down-regulating the RCOR2 level. a) WT or B7-H4-null $YFP^+Mac\text{-}1^+c\text{-}Kit^+$ LICs of primary recipients were subjected to mRNA-sequencing. Gene ontology (GO) analysis of biological process is shown, and the candidate changes in "transcription, chromosome organization or chromatin organization" are highlighted in red. b) Heat-map of significantly changed genes from mRNA-sequencing analysis in panel a. c) mRNA-sequencing results for the potential candidate genes related to transcription factors, myeloid differentiation and self-renewal. d) RCOR2 levels were validated in WT cells, B7-H4-null cells and Rcor2-knockdown B7-H4-null cells from the rescue experiment.

sFigure 5

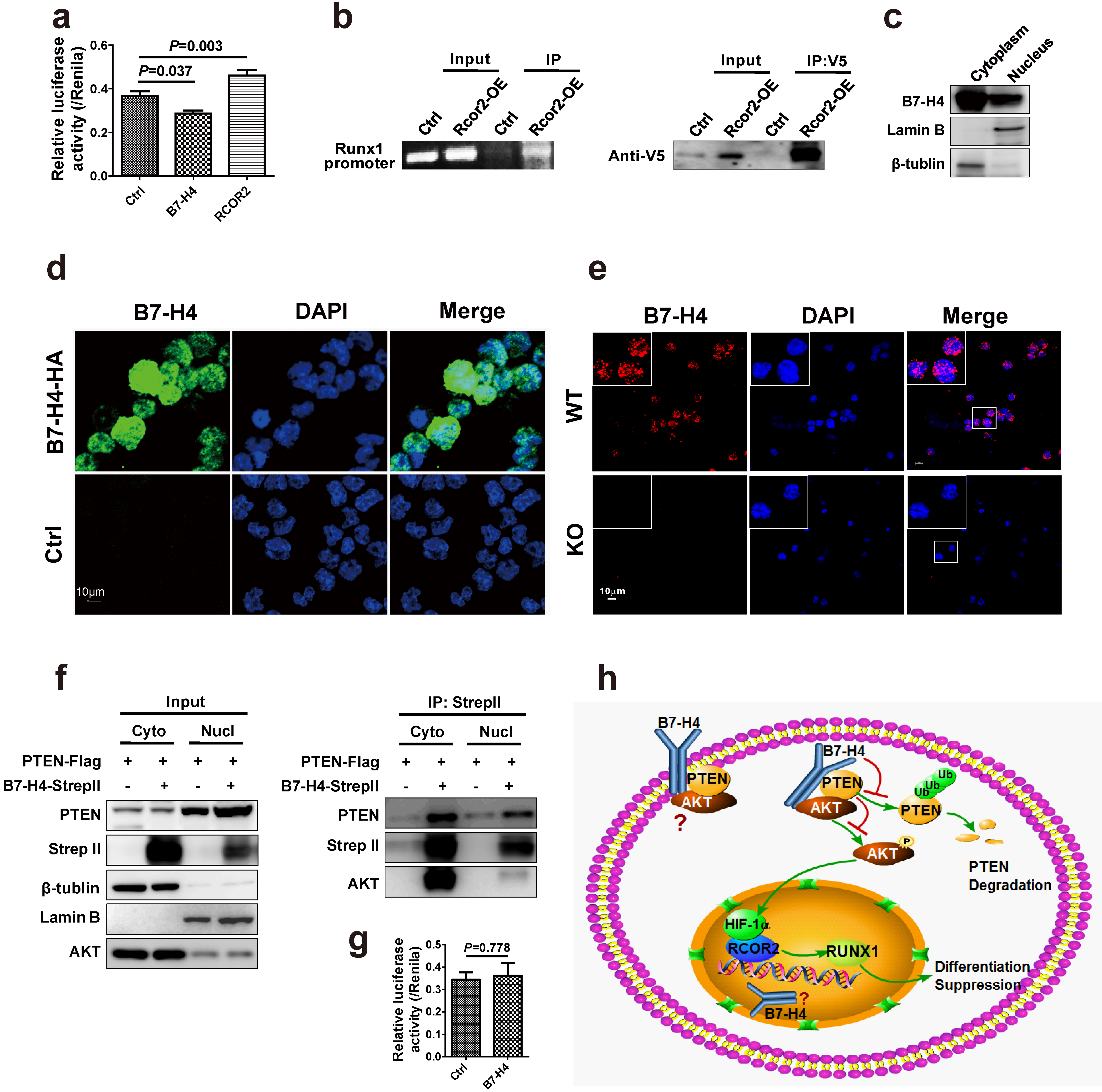
a
Relative luciferase activity (/Renila)
0.0
0.2
0.4
0.6
P=0.003
P=0.037
Ctrl
B7-H4
RCOR2
b
Input
IP
Ctrl
Rcor2-OE
Ctrl
Rcor2-OE
Runx1 promoter
Input
IP:V5
Ctrl
Rcor2-OE
Ctrl
Rcor2-OE
Anti-V5
c
Cytoplasm
Nucleus
B7-H4
Lamin B
β-tublin
d
B7-H4
DAPI
Merge
B7-H4-HA
Ctrl
10μm
e
B7-H4
DAPI
Merge
WT
KO
10μm
f
Input
Cyto
Nucl
PTEN-Flag + + + +
B7-H4-StrepII - + - +
PTEN
Strep II
β-tublin
Lamin B
AKT
IP: StrepII
Cyto
Nucl
PTEN-Flag + + + +
B7-H4-StrepII - + - +
PTEN
Strep II
AKT
g
Relative luciferase activity (/Renila)
0.0
0.1
0.2
0.3
0.4
0.5
P=0.778
Ctrl
B7-H4
h
B7-H4
PTEN
AKT
?
B7-H4
PTEN
AKT
Ub
Ub
Ub
PTEN
P
AKT
PTEN
Degradation
HIF-1α
RCOR2
RUNX1
Differentiation
Suppression
?
B7-H4

sFigure 5. B7-H4 suppresses RCOR2 to reduce RUNX1 expression through PTEN/AKT signaling. a) Luciferase activity was measured in 293T cells containing a Runx1 luciferase reporter at 24 h following the over-expression of B7-H4 or Rcor2, experiments were performed in sextuple. b) ChIP assays were performed in 293T cells transfected with pLX304-Rcor2-V5 plasmid or empty vector (Ctrl). PCR products showing the input control and the amplification of the Rcor2-binding sequence of Runx1 were measured by semi-quantitative PCR (left panel). Immunoprecipitated fractions were validated by the anti-V5 (RCOR2) antibodies using immunoblotting (right panel). c) Cytoplasmic and nuclear forms of B7-H4 were evaluated in B7-H4-over-expressing 293T cells using western blotting. Laminin-B and β-tublin were used as controls for nuclear and cytoplasmic extracts, respectively. d-e) B7-H4 was examined in B7-H4-over-expressing C1498 cells (green) or mouse primary AML cells (red) using immunofluorescence staining as determined by a confocal microscope. Nucleus were stained by DIPA (blue) f) StrepII-tagged B7-H4 and Flag-tagged PTEN were over-expressed in 293T cells, and their lysates were fractionated for cytoplasmic and nuclear portions, followed by the co-immunoprecipitation using Strep-tactin Sepharose and western blotting analysis for Flag (PTEN) and endogenous levels of AKT. g) Luciferase activity was measured in 293T cells containing a PTEN luciferase reporter 24 h following the over-expression of B7-H4, experiments were performed in sextuple. h) Schematic diagram of the functions of B7-H4 in leukemogenesis.
